# Successful Leadless Pacemaker Implantation After Lead Extraction in a Patient With Ventricular Septal Defect Patch

**DOI:** 10.1002/joa3.70231

**Published:** 2025-11-24

**Authors:** Keigo Misonou, Michio Nagashima, Hiroyuki Kono, Koumei Onuki, Maiko Kuroda, Jun Hirokami, Rei Kuji, Tomonori Katsuki, Kengo Korai, Masato Fukunaga, Kenichi Hiroshima, Kenji Ando

**Affiliations:** ^1^ Department of Cardiology Kokura Memorial Hospital Fukuoka Japan

**Keywords:** Aveir, congenital heart disease, device infection, lead extraction, leadless pacemaker, ventricular septal defect

## Abstract

**Introduction:**

Lead extraction and reimplantation in adult congenital heart disease (CHD) patients is challenging due to anatomical complexity.

**Case:**

A 76‐year‐old man with prior ventricular septal defect (VSD) patch repair and pacemaker implantation developed a device infection. Complete transvenous lead extraction (TLE) was achieved using laser and mechanical sheaths. A leadless pacemaker (Aveir VR) was implanted at the right ventricular outflow tract (RVOT) using pre‐fixation mapping to avoid the VSD patch.

**Conclusion:**

This case illustrates the effectiveness of pre‐mapping in achieving safe reimplantation of a leadless pacemaker after TLE in complex CHD anatomy.

## Graphical abstract

This report describes successful transvenous lead extraction followed by leadless pacemaker (Aveir VR) implantation in a 76‐year‐old man with a repaired VSD and chronic device infection. Dense intravascular adhesions required combined laser and mechanical sheath techniques for complete lead removal. Pre‐fixation mapping enabled safe deployment of the leadless pacemaker while avoiding the calcified VSD patch, demonstrating the feasibility of this strategy in complex congenital heart disease cases.

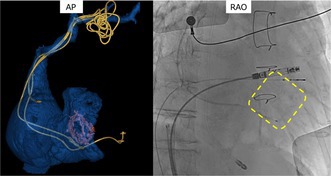

## 1 Introduction

Lead‐ and pacemaker pocket‐related complications are of relevance in the adult congenital heart disease (CHD) population. Reports from pediatric and CHD centers have demonstrated that transvenous lead extraction (TLE) in patients with CHD is safe and has a high success rate. However, younger age at initial device implantation and older lead age at the time of extraction have been identified as risk factors for complex extractions [1]. In addition, reports on leadless pacemaker implantation in CHD patients are limited [2]. In this report, we describe a case of TLE and leadless pacemaker (Aveir VR [Abbott, Chicago, USA]) implantation in a patient who had undergone patch repair for a ventricular septal defect (VSD) and pacemaker implantation for complete atrioventricular block (CAVB) 38 years earlier.

## 2 Case

**FIGURE 1:**
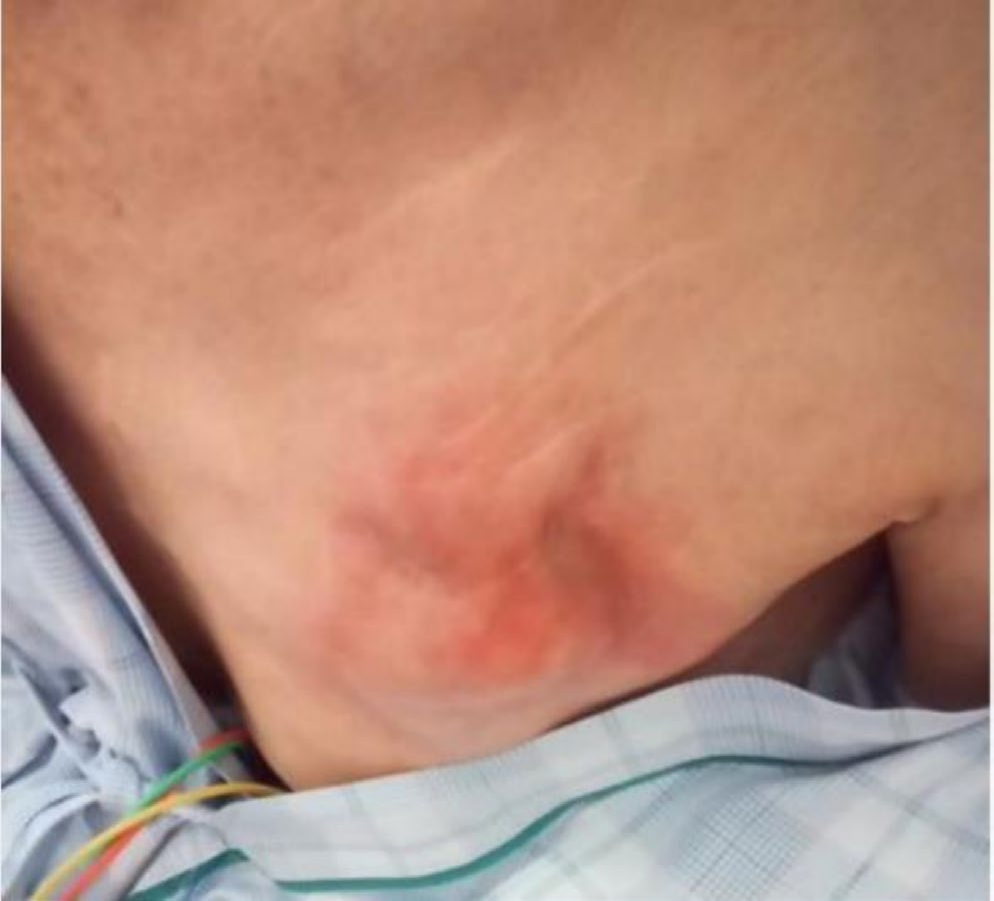
Illustrates the CRT‐P generator pocket. The pocket shows redness and swelling.

The patient was a 76‐year‐old man who had undergone patch repair for a large VSD 38 years earlier. Following the surgery, he developed CAVB and received a transvenous pacemaker system implanted in the left prepectoral region. Ten years ago, he experienced heart failure and underwent an upgrade to a cardiac resynchronization therapy pacemaker (CRT‐P). Three months prior to referral, he underwent CRT generator replacement—the eighth generator replacement in total. Postoperatively, a hematoma was noted but was initially monitored due to a resolving trend. However, approximately 1 week prior to referral, erythema, swelling, and warmth at the device pocket worsened. He presented to his previous hospital, where he was diagnosed with a device infection and subsequently transferred to our institution for lead extraction. Physical examination revealed erythema, swelling, and warmth at the pocket site (Figure 1).

**FIGURE 2:**
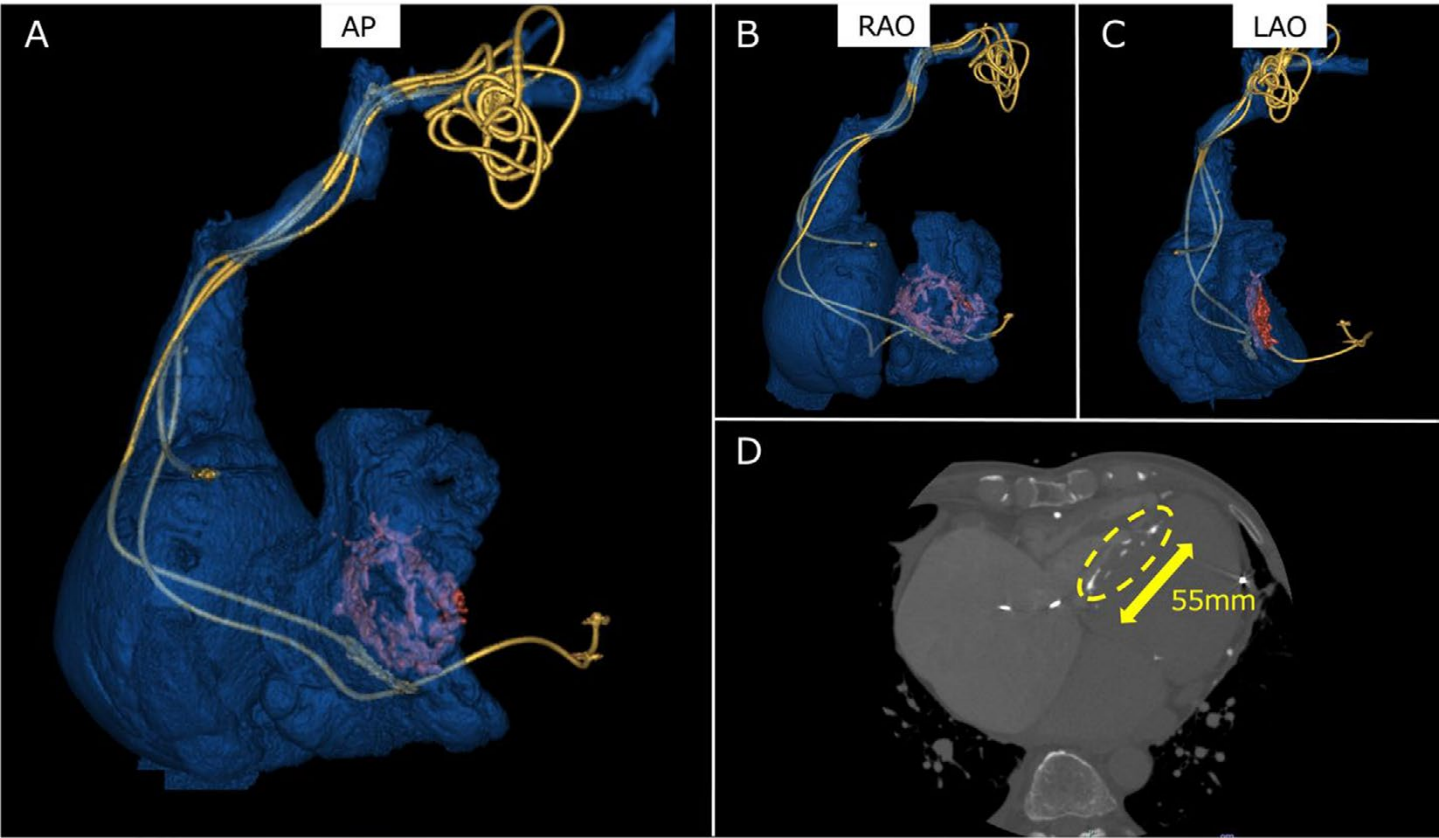
(A) Anteroposterior (AP), (B) right anterior oblique (RAO), and (C) left anterior oblique (LAO) views of 3D reconstructed CT images showing the course of the lead; the orange‐colored area represents the VSD patch. (D) Axial CT image demonstrating the VSD patch with surrounding calcification, indicated by the yellow dotted line. The maximum diameter of the patch is 55 mm.

Laboratory testing showed a white blood cell count of 8200/μL and a C‐reactive protein (CRP) level of 0.1 mg/dL. Subsequent blood cultures were negative. A 12‐lead electrocardiogram demonstrated atrial fibrillation with biventricular pacing at a rate of 60 bpm. Echocardiographic findings were as follows: left ventricular (LV) ejection fraction 33%, LV end‐diastolic diameter 68 mm, LV end‐systolic diameter 57 mm, left atrial diameter 66 mm, moderate tricuspid regurgitation, and mild–moderate mitral regurgitation. The right atrial (RA) lead was fractured, and the device was operating in VVI mode. In preparation for lead extraction, contrast‐enhanced computed tomography (CT) was performed to delineate lead course and the anatomical location of the VSD patch. CT imaging revealed that the right ventricular (RV) lead was positioned at the RV inferior wall, LV lead was positioned at the lateral vein, the RA lead was located in the RA appendage, and the VSD patch—measuring 55 mm in its longest dimension—was located from the ventricular base to the mid septum (Figure 2).

## 3 Methods

Given the prolonged implant duration and potential for significant adhesions, extraction was planned under general anesthesia with transesophageal echocardiographic (TEE) monitoring. From the right femoral vein, a 10‐Fr sheath was introduced for intracardiac echocardiography and balloon occlusion if needed, a 5‐Fr sheath for temporary pacing, and an additional 4‐Fr sheath for arterial pressure monitoring. The patient was taking edoxaban 30 mg for atrial fibrillation. Anticoagulation was withheld only on the morning of the procedure and was resumed the following morning. The left prepectoral pocket exhibited partial purulent discharge and poor granulation tissue, and surgical debridement was performed. The leads could not be removed by simple traction; therefore, locking stylets were inserted, and sequential extraction of the LV, RV, and RA leads was attempted using the GlideLight laser sheath (Philips, Amsterdam, Netherlands). Due to dense adhesions, laser sheath extraction alone was insufficient, and mechanical extraction using the Evolution RL sheath (Merit Medical, South Jordan, USA) was employed, leading to the successful removal of all leads. As no intrinsic rhythm was observed, a temporary pacing lead was placed via the right internal jugular vein, and the patient was transferred to the general ward. However, shortly after transfer, body movement caused pacing failure, leading to cardiac arrest. Cardiopulmonary resuscitation was initiated, including transcutaneous pacing. A new temporary pacing lead was inserted via the left femoral vein, and the previously placed right internal jugular lead was repositioned, resulting in satisfactory sensing and capture thresholds.

**FIGURE 3:**
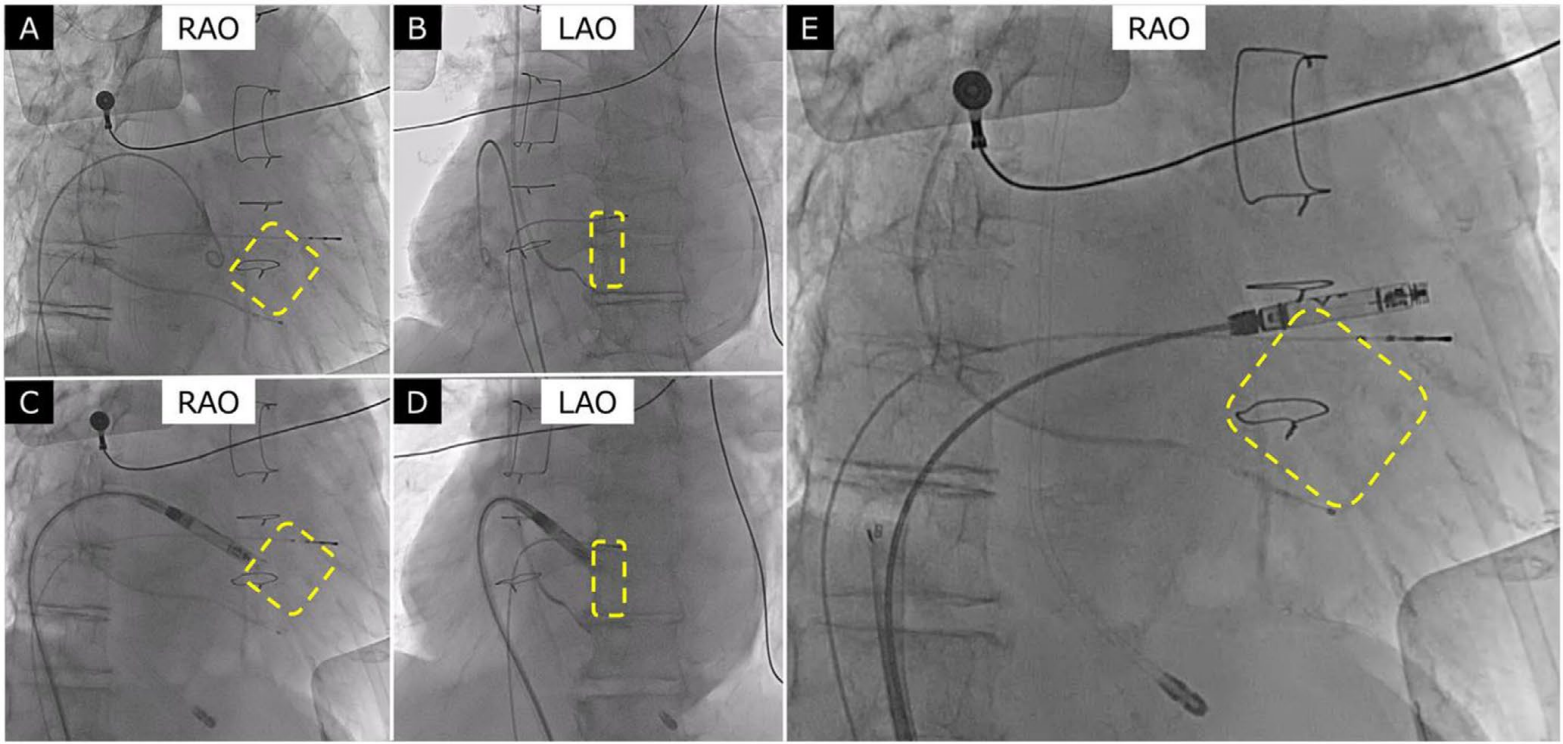
In panels A–E, the calcified VSD patch is clearly indicated by a yellow dotted outline. (A) RAO and (B) LAO views of contrast imaging of the right atrium and right ventricle, demonstrating marked RA enlargement. (C) RAO and (D) LAO views showing the tip of the Aveir VR leadless pacemaker positioned near the VSD patch, where pacing capture could not be achieved. (E) Final implantation site of the Aveir VR in the right ventricular outflow tract (RVOT).

**FIGURE 4:**
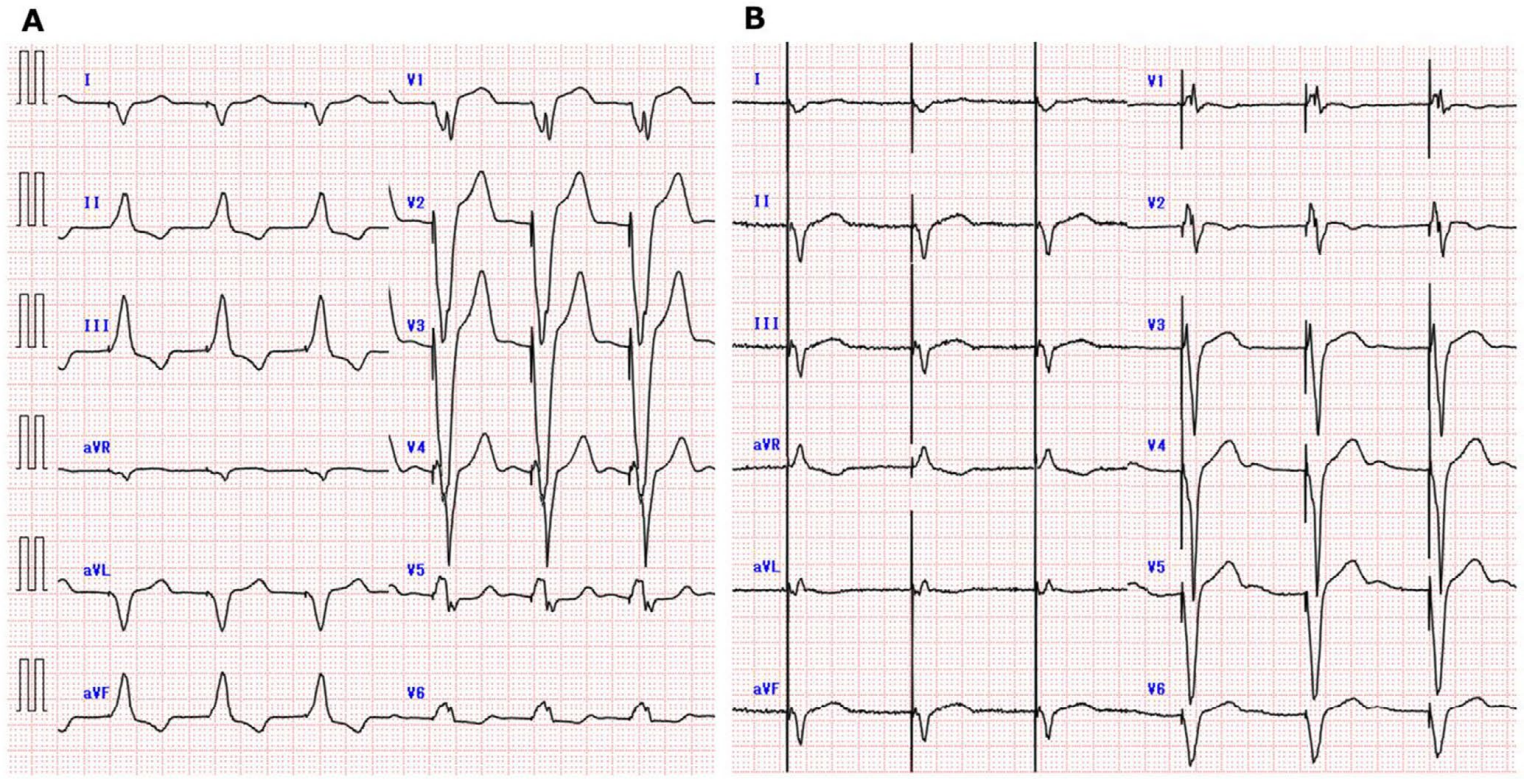
Representative 12‐lead electrocardiograms. (A) Pre‐extraction of CRT‐P: Atrial fibrillation with biventricular pacing. (B) Post‐implantation of Aveir VR: VVI pacing from the leadless pacemaker.

On postoperative day 2, negative blood cultures were confirmed, and a decision was made to implant a leadless pacemaker (Aveir VR) via the right femoral vein. RA and RV angiography revealed significant RA enlargement. Calcification around the VSD patch was noted, allowing identification of the patch location under fluoroscopy (Figure 3). Pre‐fixation mapping at the calcified VSD patch and its surrounding septal tissue failed to achieve capture. Pre‐fixation mapping at the right ventricular outflow tract (RVOT) septum demonstrated adequate signal amplitude and pacing thresholds. The device was successfully fixed by screwing it into position and deployed without complications. The temporary pacing leads from both the right internal jugular and right femoral veins were removed, concluding the procedure. The final implant parameters were as follows: R‐wave amplitude was not measurable, as the patient had no intrinsic ventricular rhythm; pacing threshold was 0.75 V/0.4 ms; impedance was 1060 Ω; final programmed output was 3.5 V/0.4 ms; and projected battery longevity was 12.1 years. At one‐month follow‐up, pacing threshold remained stable at 0.25 V/0.4 ms, impedance was 840 Ω, and R‐wave amplitude was again not measurable due to the absence of intrinsic ventricular rhythm. No complications or reinfection occurred. The 12‐lead electrocardiograms are shown in Figure 4, demonstrating a QRS duration of 166 ms before extraction and 200 ms after leadless pacemaker implantation.

## 4 Discussion

TLE in patients with CHD presents unique anatomical and procedural challenges, particularly in individuals with a long duration since initial device implantation. In this case, the patient had undergone VSD patch repair and pacemaker implantation 38 years earlier, with subsequent CRT upgrade and multiple generator replacements. Chronicity of lead implantation and prior cardiac surgery both likely contributed to dense intravascular and intracardiac adhesions, necessitating the use of both laser and mechanical sheaths to achieve successful lead removal.

Device‐related infections in CHD patients necessitate complete system removal [3], and lead age and younger age at initial implantation have been identified as predictors of complex extraction procedures [1]. In our case, thorough preprocedural planning, including contrast‐enhanced CT and intraoperative TEE monitoring, enabled safe extraction under general anesthesia. Despite the challenging anatomy and lead adhesions, complete removal was achieved without vascular or cardiac injury.

Reimplantation strategy following lead extraction in CHD patients should consider prior infections, limited venous access, and altered intracardiac anatomy. Leadless pacing systems have emerged as an attractive alternative in such scenarios, avoiding leads and subcutaneous pockets, which are common sites of infection and mechanical complications [4]. There has been a report of leadless pacemaker implantation following lead extraction for device infection, with no reinfection observed during a 2‐year follow‐up [5]. However, reports of leadless pacemaker implantation following lead extraction in patients with CHD remain very limited, and available data are scarce. A previous report described the implantation of a leadless pacemaker (Micra, Medtronic, Minneapolis, USA) after interventional VSD closure [6]. In this case, the Aveir VR system was selected for its ability to perform pre‐fixation mapping [7]. This feature allowed us to evaluate pacing and sensing thresholds before final deployment, enabling us to deliberately avoid the calcified VSD patch visualized under fluoroscopy. Although pre‐fixation mapping at the VSD patch and its surrounding septal tissue failed to produce adequate pacing parameters, pre‐fixation mapping at the RVOT septum demonstrated satisfactory signal amplitude and pacing thresholds. Consequently, the device was successfully fixed in the RVOT without complications. This patient had undergone CRT‐P implantation 10 years earlier with only a limited response, showing persistent heart failure symptoms and no significant improvement in LVEF. Given the patient's advanced age, frailty, and recurrent device‐related complications, we prioritized infection control and procedural safety in the current setting. Therefore, re‐implantation of a CRT‐P system was deemed unsuitable, and a leadless pacemaker was selected as the safest available option to ensure reliable pacing while minimizing further infection risk. In addition, the Aveir VR is a retrievable device, and if heart failure worsens due to RV pacing in the future, device retrieval and reimplantation of CRT on the contralateral side may be considered.

## 5 Conclusion

This case demonstrates the feasibility and safety of TLE followed by leadless pacemaker implantation in a complex CHD patient with a surgically repaired large VSD nearly four decades prior. The ability to perform pre‐fixation mapping with the Aveir VR system enabled optimal device placement while avoiding the VSD patch. Careful imaging, procedural planning, and technology selection are key to successful outcomes in this population.

## Funding

The authors have nothing to report.

## Consent

The patient has provided consent for publication.

## Conflicts of Interest

The authors declare no conflicts of interest.

## Data Availability

The authors have nothing to report.
